# AnimalTraits - a curated animal trait database for body mass, metabolic rate and brain size

**DOI:** 10.1038/s41597-022-01364-9

**Published:** 2022-06-02

**Authors:** Marie E. Herberstein, Donald James McLean, Elizabeth Lowe, Jonas O. Wolff, Md Kawsar Khan, Kaitlyn Smith, Andrew P. Allen, Matthew Bulbert, Bruno A. Buzatto, Mark D. B. Eldridge, Daniel Falster, Laura Fernandez Winzer, Simon C. Griffith, Joshua S. Madin, Ajay Narendra, Mark Westoby, Martin J. Whiting, Ian J. Wright, Alexandra J. R. Carthey

**Affiliations:** 1 School of Natural Sciences, Macquarie University, Sydney, Australia; 2 Zoological Institute and Museum, University of Greifswald, Greifswald, Germany; 3 Department of Biological and Medical Sciences, Oxford Brookes University, Oxford, United Kingdom; 4 College of Science and Engineering, Flinders University, Adelaide, Australia; 5 Australian Museum Research Institute, Australian Museum, Sydney, Australia; 6 Evolution & Ecology Research Centre, University of New South Wales, Sydney, Australia; 7 Hawai’i Institute of Marine Biology, University of Hawai’i at Mānoa, Kāne’ohe, HI USA; 8 Hawkesbury Institute for the Environment, Western Sydney University, Sydney, Australia

**Keywords:** Behavioural ecology, Evolution

## Abstract

Trait databases have become important resources for large-scale comparative studies in ecology and evolution. Here we introduce the AnimalTraits database, a curated database of body mass, metabolic rate and brain size, in standardised units, for terrestrial animals. The database has broad taxonomic breadth, including tetrapods, arthropods, molluscs and annelids from almost 2000 species and 1000 genera. All data recorded in the database are sourced from their original empirical publication, and the original metrics and measurements are included with each record. This allows for subsequent data transformations as required. We have included rich metadata to allow users to filter the dataset. The additional R scripts we provide will assist researchers with aggregating standardised observations into species-level trait values. Our goals are to provide this resource without restrictions, to keep the AnimalTraits database current, and to grow the number of relevant traits in the future.

## Abstract (summary)

**Table:** 

Measurement(s)	metabolic rate quantification • body mass • brain size
Technology Type(s)	metabolic rate measurement • body mass quantification • brain mass brain volume

## Background & Summary

Large, multi-species trait-based comparative approaches have been successfully applied in animal and plant functional ecology, comparative physiology and macroevolution^1–4^, and bacterial and archaeal traits^5,6^. More recently, there have been calls to establish more taxon-specific databases^7^. Trait databases provide a major boost to this research by concentrating scattered trait data into one central repository. For example, the quantification of functional diversity in 46,000 vascular plants enabled the generation of a global trait space into which plant lineages and plant communities were mapped^8^. Trait databases also have application to conservation. For example, a trait database of corals was used to better understand the mechanisms associated with coral bleaching^9^.

The disciplines of animal physiology, ecology and behaviour have a long tradition of collating large volumes of trait data into meta-analyses to test theoretical predictions. For example, comparative methods have been applied to ask questions about insect body size variation^2^, the relationship between reptile brain size, sociality, and environmental complexity^10^, the phenological response of animals to climate change^11^, and variation in metabolic rate across all domains of life^12^. These databases are usually static and have been amassed with very specific questions in mind. They often include some form of data transformation from the original published data and may incorporate previously published data sets that have also applied various data transformations. These approaches limit the future usefulness of these data compilations because the ability to transform original raw data into alternative metrics carries data integrity risks that are difficult - if not impossible - to detect and correct.

Here, we introduce a curated animal trait database that includes three basic functional traits: body size, metabolic rate, and brain size, for terrestrial animals. Each row in the database is an ‘observation’; one or more trait values measured from a single specimen or group of specimens of the same species. These observations can be aggregated into species-mean trait values using the R script we provide. We have limited our database to these initial traits because they are central to many ecological questions and in order to prioritize exceptionally ‘clean’ data. We intend to continue to expand the number of included traits with time. The distinctive value of this new animal trait database is four-fold:

1. Open access: the data are openly available to researchers without restrictions^13^;
2. Taxonomic breadth: the database includes a broad taxonomic range of terrestrial animal species including several groups of tetrapods and arthropods, as well as molluscs and annelids;
3. Clean, empirical data: all data are sourced from the original publication that made and reported on the included measurements, and are entered into the database using the original metrics – all subsequent transformations can be applied to these original data, meaning it is eminently reusable by future researchers;
4. Annotation: we have included useful methodological metadata (such as measurement method and parameters) that allow researchers to filter the dataset as needed.

While the AnimalTraits database is relatively small compared to other studies that have amassed data on the same or similar traits, its distinguishing feature is that it contains high quality raw data. Raw data are often no longer available from published databases that might only include species mean values based on unknown sample sizes, often even single datapoints. Moreover, the AnimalTraits database also includes the sample size of means or ranges when those were available from the original papers. This allows the user to exercise ultimate control over data selection.

## Methods

Our goal was to generate a reliable and high-quality trait database with ultimate transparency and flexibility, while minimising error prone manual or *ad hoc* data conversions. For example, some data compilations only report converted data (e.g. watts for metabolic rates), without providing the raw data and the conversion equation. This not only limits the utility of the data, as they cannot be converted back to their original form, but it also prevents any form of quality control associated with converting the data.

Our database compilation process consisted of the following steps:

1. Data selection: identifying sources of trait data from peer reviewed papers.
2. Transcription: manually extracting the data and transcribing them into a comma-separated values (CSV) file (a ‘raw’ data file), retaining the measurement units as published.
3. Standardisation: programmatically reading all raw data files, converting to standard units for each trait, performing data validity checks, and combining into a single CSV file of standardised observations (performed in R, scripts available in auxiliary material).
4. Quality control: performing additional data quality checks (see below) on the standardised observations; correcting any processing errors and excluding problematic data from the database.

To build the trait database, we collected measurements of body mass, brain size and metabolic rate from published, peer-reviewed data sources. Not all three variables had to be reported in the original source to be included in the database. Any units of body mass or weight were acceptable for recording body mass in the raw files. Brain sizes were recorded as either volume or mass. We accepted raw metabolic rates expressed as rate of CO_2_ production, rate of O_2_ consumption, or rate of energy transfer, i.e. power measured in watts or joules/sec. Both mass-specific metabolic rate (consumption of energy per gram of body mass per unit of time) and whole-body metabolic rate (consumption of energy for the whole body per unit of time) were recorded from the source data. Mass-specific metabolic rate was converted to whole-body metabolic rate (or *vice versa*) when the data source provided a value for body mass. We further recorded the method used to measure metabolic rate, e.g. basal or resting metabolic rate (metabolic rate of an inactive, fasting animal in its thermal neutral zone), standard metabolic rate (resting metabolic rate of an ectotherm), or field metabolic rate (average respiration rates of free living animals). In the database (‘metabolic rate- method’ column), we recorded the type of metabolic rate measured as specified by the original researchers.

We created one raw file for each source data publication (Table 1), with all observations transcribed into a predefined set of columns (raw data columns are described in the Template.xslx spreadsheet in the auxiliary material). Each row contained a single trait measurement. Each measured entity (i.e. an animal or a group of animals measured together) was assigned an object identifier that was unique within the raw file. This meant that when multiple traits were measured for a single entity (such as both body mass and metabolic rate), those measurements shared the same object identifier. To minimise transcription or unit conversion errors, values were transcribed into the raw file as originally reported in the source document, along with the reported units. When a paper described multiple treatment groups, we collected data from the control or the ‘least manipulated’ group. We also recorded the temperature at which metabolic rate was recorded and the applicable respiratory quotient (if reported).

**Table 1:** Summary of the traits contained in the Animal Trait Database and the primary source for the data.

Trait	Units	Observation count	Species count	Reference
Metabolic rate	Watts (W)	1185	662	^21–192^
Body mass	Kilograms (kg)	2856	1830	^19,21–152,154–213^
Brain mass	Grams (g)	2361	1445	^19,193–237^

Whenever possible, we collected traits measured from a single individual; however, we also allowed mean values with sample sizes or a range of values to be specified as ‘min – max’ (together with a sample size). During standardisation, ranges were reduced to their midpoints, although the minimum and maximum values were also standardised and recorded in the database. In the current release of the database, two data sources (49 observations) specified a range for body mass, and no other traits were specified as range. If a user prefers not to use range reduction to midpoint for an application, such rows can be manually filtered from the database before use by selecting non-empty minimum or maximum values. Measurement units allowed optional additional information within round brackets, e.g. ‘(CO2) ml’ or ‘(O2) l’ for metabolic rate measured as either millilitres of CO_2_ produced or litres of O_2_ consumed, respectively.

### Standardisation

The standardisation step consisted of compiling R scripts^14^ that read the raw data CSV files, performed various checks, transformed the values into standard units, then wrote the result to a single CSV file of standardised observations. We included the following checks: trait type was ‘body mass’, ‘metabolic rate’, ‘mass-specific metabolic rate’ or ‘brain size’; units were known and interpretable (detail below); and scientific names were defined by the R package *taxize*^15^. In addition, we checked the binomial names against several databases (see auxiliary material) and corrected any flagged spelling errors. We updated any binomial name changes that we were aware of and encourage readers to contact us with further updates.

The automated conversion process was implemented to avoid a class of errors potentially introduced by manual conversion of values during data entry. Preserving the source units makes it simple to double-check for transcription errors by comparing the transcribed raw data files with the source data. Base units were converted using the R packages *units*^16^ and *udunits2*^17^. Some traits required additional, non-standard unit conversion handling. Units sometimes contained a numeric factor (prefixed by ‘x’), which was multiplied with the trait value (e.g. ‘x6.4e-5 mm^3^’) during the standardisation step.

We converted raw units of body mass to kilograms during the standardisation process. Mass and volume measures of brain sizes were standardised to mass in kilograms. While users can apply their own statistics to convert brain volume to mass, we included this step as an additional service to users applying published conversion metrics with an assumed density of 1.036 g/mL e.g.^18,19^. This conversion factor is based on gravity measurements of fresh vertebrate brain tissue.

All metabolic rates were converted to watts. To achieve this, CO_2_ production was first converted to the equivalent consumption of O_2_. This conversion was only possible when the respiratory quotient (*q*) was specified in the original data source and recorded in the raw CSV file. Conversion of CO_2_ production to O_2_ consumption used the equation

1 $${O}_{2}=C{O}_{2}/q$$

Rate of O_2_ consumption was converted to watts by multiplying by a conversion factor of 20 J/ml^12^. Non-endothermic metabolic rates, *q*, measured at a temperature other than 25 °C were transformed to their equivalent at 25 °C, *q*_25_, with the equation

2 $${{\rm{q}}}_{25}={\rm{q}}\times {Q}_{10}^{(25-T)/1{0}^{\circ }C}$$

where Q_10_ is the temperature coefficient; the factor by which the metabolic rate changes for each change of 10 temperature units. We used Q_10_ = 2.21 or 2.44 for amphibians and reptiles respectively, and 2 for all other non-endothermic taxa (which were arachnids, insects, crustaceans and myriapods)^12^. Q_10_ values can be updated in light of more specific values becoming available in future. The standardisation parameters follow Makarieva *et al*.^12^, however they can be modified and the database recompiled by users wishing to use different parameters or output units (see Usage Notes).

Both mass-specific and whole-body metabolic rate were handled by the standardisation process. Mass-specific metabolic rate was converted to whole-body metabolic rate (and *vice versa*) if the observation also included a value for body mass.

## Data Records

As we grow this database, new versions will be released along with any corrections.

**Fig. 1:**
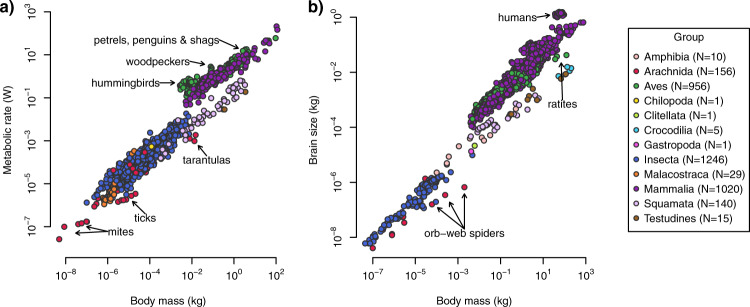
Overview of the ranges of trait values in the database at time of publication. Each point represents a single observation of (**a**) body mass and metabolic rate, and (**b**) body mass and brain size. To orient the reader, some taxa with outstanding trait values are labelled in the graph. The differing allometries of endotherms and ectotherms are apparent for both metabolic rate and brain size. Axes are log-scaled.

At the time of publication, the animal traits database contained over 3500 observations from over 200 data sources. The almost 2000 terrestrial species in the database came from over 1000 genera, 350 families, 90 orders and four phyla (Chordata, Arthropoda, Annelida, Mollusca). For the majority of species in the database, we transcribed body mass data (>1700 species) and brain size data (>1400 species), with both traits recorded for over 1200 species (Table 1). The database contains metabolic rate data for over 600 species. Body mass measurements span 10 orders of magnitude (Fig. 1), while metabolic rates and brain sizes both span 8 orders of magnitude (Fig. 1). Details of trait data are summarised in Table 1 along with the primary sources, which should be referenced wherever possible.

The database is under a CC0 1.0 licence with unrestricted use. It consists of a single CSV file (docs/observations.csv in the online auxiliary material on Zenodo^20^ (also under a CCO 1.0 licence) or accessible from the website https://animaltraits.org) The database columns are described in the online file docs/column-documentation.csv. We also provide an Excel spreadsheet that contains both the database content and the column descriptions as separate worksheets (docs/observations.xlsx online or downloadable from the website). The auxiliary material includes all raw CSV data files (located within the data/raw folder) and the R scripts used to compile and standardise the raw data into the final database (located within the R folder), as well as the source for the website (in the docs folder). Text files named README.md in most folders describe the auxiliary material in greater detail.

The database contains one row per observation. It includes columns that describe the specimen taxa, sex (when specified), sample size, full data source reference and optional comments. Each trait has columns for the standardised trait value and units, the original trait value and units, and the (standardised) minimum and maximum values for ranges. Additional columns include the temperature at which metabolic rate was recorded and the respiratory quotient used to convert from CO_2_ production to the equivalent O_2_ consumption. Metadata columns for metabolic rate apply to both metabolic rate and mass-specific metabolic rate. The columns are fully documented in the auxiliary material.

We provide R scripts to assist with aggregating standardised observations into species-level trait values (see below, Code Availability). We provide a script rather than a standard species-traits data set, in order to provide flexibility to customise the aggregation as needed. In this way, researchers have full control over both the records to be combined and the way in which they are combined. For example, a researcher might choose to exclude body mass observations recorded as a range, and aggregate males and females separately.

## Technical Validation

We included only peer reviewed data sources that reported original observations (thus not from reviews, meta-analyses or existing databases or data compilations) on terrestrial animals identified to genus or species. Data sources that were difficult to interpret, or from which raw data measurements could not be extracted, were excluded. We used reviews and meta-analyses to identify suitable papers from which we extracted the original data.

Our unit conversion scripts detected and reported an error for several types of data entry issues, including when the conversion script was unable to convert the units, which most likely indicates a data entry error. Furthermore, invalid, incomplete or problematic data generated warnings, including unknown taxa or metabolic rate specified as CO_2_ production with no respiratory quotient. Additional quality check steps involved plotting results and checking for outliers.

Whenever a problem was reported by the standardisation or quality check steps, we first checked if it was caused by a transcription error by comparing the raw file to the source data. If so, we fixed the raw file. Otherwise, we checked if the problem resulted from a failure of the standardisation step (i.e., a programming error). If it did, we fixed the standardisation scripts, otherwise we checked if an error was apparent in the source paper, such as incorrect units or a misplaced decimal place. If we found an apparent error in the source data or the observation could not be standardised, we excluded either the entire data source or else the problematic observations. Otherwise, we retained the paper and data.

## Usage Notes

This database can be used to address a number of current biological questions including how metabolic rate and brain size scale to body size in broad taxonomic groups. More focused questions can be addressed by combining it with additional species-specific data such as behaviour, distribution or range limits, life history tactics or pace of life, or phylogenetic and genomic data. Advanced users are able to use the supplied R scripts to compile and standardise the database using different standardisation parameters or output units; see the online file R/README.md for more details. Finally, as we have included metadata on the methods for obtaining metabolic rate and brain size, the impact of method bias on rate and size estimate can be explored. The database is available in the auxiliary material^20^, and it can be download as either a UTF-8 encoded CSV file or a Microsoft Excel spreadsheet file from https://animaltraits.org.

### Access

The static version of the dataset described here is available via Zenodo^20^ under a CC0 1.0 licence, which allows for the unrestricted use of the dataset. We kindly ask that users of the database cite this descriptor and the original sources cited within this descriptor.

## Data Availability

The observations database, all raw CSV files and the R scripts used to standardise and check the observations, as well as a sample script to aggregate the observations database into a species-trait data set are available in the auxiliary material^20^. The auxiliary material also contains README.txt files that describe the structure and usage of the data and scripts. The auxiliary material is managed as a GitHub repository (https://github.com/animaltraits/animaltraits.github.io). GitHub is also used to build and serve the website.
